# The Effect of Apnea on Central Airway Oxygen Concentration During Rigid Bronchoscopy: A Prospective Observational Study

**DOI:** 10.7759/cureus.86297

**Published:** 2025-06-18

**Authors:** Joseph C Keenan, Jennifer L Wong, H Erhan Dincer, Alexander Kaizer, Roy J Cho, Sudarshan Setty

**Affiliations:** 1 Division of Pulmonary Allergy, Critical Care, and Sleep Medicine, University of Minnesota, Minneapolis, USA; 2 Department of Biostatistic and Informatics, Colorado School of Public Health, University of Colorado-Anschutz Medical Campus, Aurora, USA; 3 Department of Anesthesia, University of Minnesota, Minneapolis, USA

**Keywords:** bronchial stenosis, central airway obstruction, jet ventilation, lung cancer, tracheal stenosis management

## Abstract

Background

Airway surgery utilizing a heat source carries a risk of airway fire. Among the airway fire triad of oxidizer, fuel, and ignition source, oxygen concentration is a modifiable risk factor. Rigid bronchoscopy, commonly used during airway surgery, utilizes an open circuit. An open circuit, when combined with jet ventilation, makes measurement of airway oxygen concentration difficult. To decrease airway oxygen concentration, some centers use a pause in jet ventilation to allow airway concentration to decrease; however, the effect of this pause on central airway oxygen levels is not known. Our objective was to better understand changes in central airway oxygen concentration during apnea during rigid bronchoscopy, an important component of fire risk.

Methods

We designed a prospective observational study of patients requiring rigid bronchoscopy. We utilized jet ventilation with 100% FiO2. To measure central airway oxygen concentration in the distal trachea, we connected a long rigid suction cannula to an oxygen analyzer and passed it through the bronchoscope. When central airway oxygen concentration was >90%, apnea was initiated, and we measured the time required for central airway oxygen concentration to decrease to less than 40%. This was repeated for the right and left main bronchi.

Results

The average time to reach airway oxygen concentration of less than 40% was 40.9±18.1s (mean±SD), 41.9±19.5s, and 41.6±21.7s for the trachea, right main bronchus, and left main bronchus, respectively.

Conclusions

We found that after a prolonged period of apnea, many patients had central airway oxygen concentration above levels conventionally considered optimal for airway surgery. This is the first description of this method for monitoring central airway oxygen concentration.

## Introduction

The rigid bronchoscope is commonly used for airway surgery. While this allows for a broad range of rigid tools and airway interventions, it presents some challenges for monitoring and safety during the procedure.

Most anesthesia machines allow for the precise monitoring of airway gas concentration when the airway is secured with a cuffed endotracheal tube. By adjusting FiO2 and simultaneously measuring end tidal oxygen concentration [1], the anesthesia provider can control the airway oxygen concentration (AiO2). This precise monitoring is not feasible during rigid bronchoscopy due to the "open" circuit, and airway gas concentration is not routinely measured.

The triad of oxidizing agent, fuel, and ignition source is well known as the key factors in airway fire risk [2]. In the setting of airway surgery with a heat source, oxygen concentration is an important modifiable risk factor. When an endotracheal tube is used in conjunction with mechanical ventilation, the American Society of Anesthesiologists (ASA) guidelines recommend decreasing the FiO2 as much as clinically feasible [2] to prevent hypoxia. As implied by this recommendation, no AiO2 is considered safe, and no specific level is recommended. Ignition of a non-laser safe endotracheal tube can occur at an AiO2 of 21%. However, the ease of ignition significantly increases at higher AiO2 [3]. This suggests that accurate knowledge of AiO2 could reduce the risk of airway fire.

The risk of airway fire during rigid bronchoscopy is low [4,5], but it has been reported [6,7]. However, there are no specific guidelines regarding oxygen concentration and fire safety during rigid bronchoscopy. To decrease the risk of inadvertently using a heat source with AiO2 above optimal levels, a method used in clinical practice is to allow for a period of apnea prior to introducing an ignition source to the field. In a recently reported series of rigid bronchoscopies, there were no airway fires when a pause was utilized prior to airway intervention [4], but the study was not powered to detect airway fire as an outcome. The effect of apnea on AiO2 in this setting is unknown.

Given that oxygen concentration is an important fire risk and that AiO2 during rigid bronchoscopy is difficult to measure, our objective for this study was to understand the effect of apnea on AiO2 during rigid bronchoscopy. With this clinical issue in mind, we devised a novel technique for measuring AiO2 in the central airways during rigid bronchoscopy, allowing us to track changes in this variable during ventilation and apnea. To our knowledge, we are the first to conduct a study of this nature.

## Materials and methods

After obtaining approval from the University of Minnesota Institutional Review Board (approval number: STUDY00008174), we screened a total of 92 patients 18 years of age and older who were scheduled for rigid bronchoscopy from February 2020 until November 2021. Patients were excluded if they were deemed unstable from a respiratory or cardiovascular standpoint by the caring physicians (pulmonologist or anesthesiologist), had a bronchopleural fistula, or were unwilling to provide consent. A total of 33 patients met criteria for inclusion in the study. Written informed consent was obtained from all patients who were included in the study. We were able to collect a full set of data from 20 patients. Data from 13 patients were excluded: six due to the surgeon's decision, six from technical problems during data collection, and one because the AiO2 did not reach 90% prior to initiating apnea.

**Figure 1:**
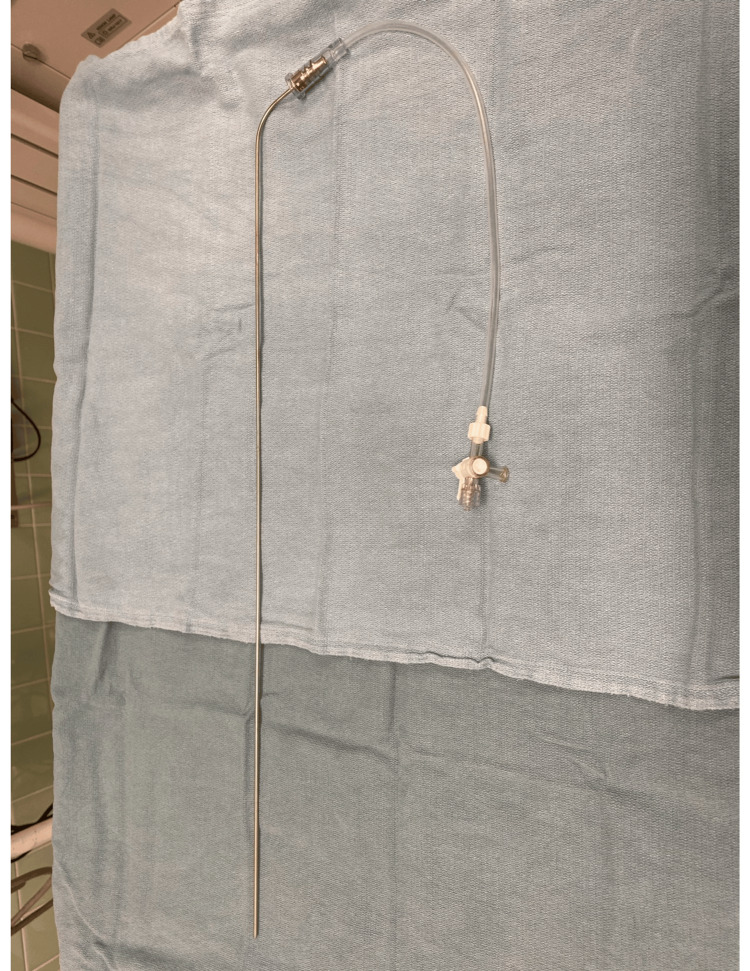
Setup of rigid suction catheter connected to a Cook Medical drainage catheter and a three-way stopcock. The gas analyzer from the anesthesia machine was connected to the three-way stopcock

To measure the AiO2, we used the gas analyzer from a Dräger Perseus A500 anesthesia machine (Lübeck, Germany). The gas sampling module of the Dräger Perseus A500 has a sidestream gas analyzer that samples gas at 200 +/- 10 ml/min. The T10-T90 is <500 msec, and the resolution of the displayed volume is 1 vol%. The gas sampling line from the anesthesia machine was hooked up to the rigid suction catheter with a Cook Medical connecting tube (Bloomington, Indiana, United States), a drainage bag connector, and a three-way stopcock (Figure 1). 

**Figure 2:**
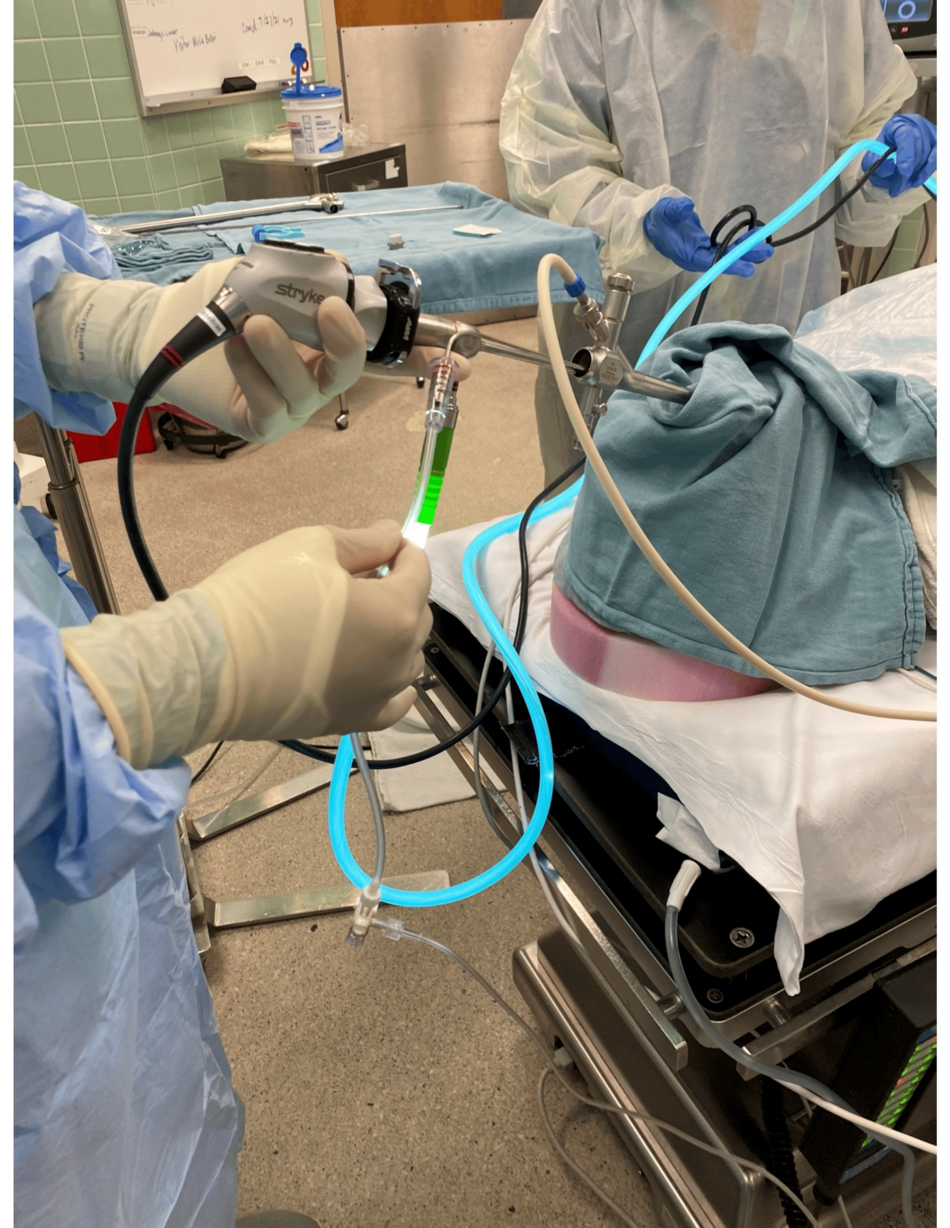
Rigid suction catheter connected to the gas analyzer being introduced through the rigid bronchoscope

**Figure 3:**
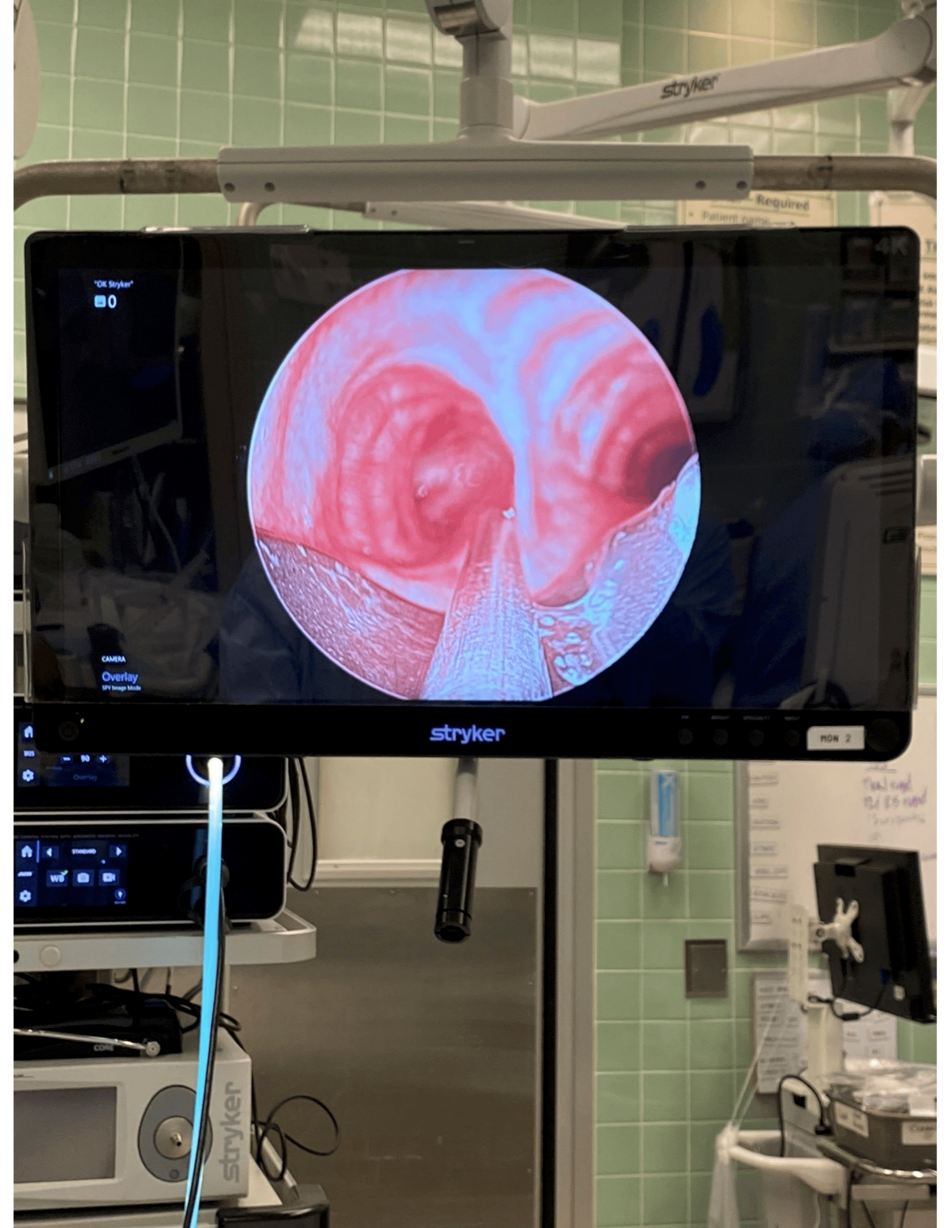
Image of rigid bronchoscope and rigid suction catheter at the level of the carina

Standard ASA monitors were applied to all patients. The choice of anesthetic agents was left to the attending anesthesiologist. After anesthesia was induced, a non-depolarizing muscle relaxant (rocuronium) was administered, and patients were ventilated by mask with FiO2 of 1.0. After the onset of neuromuscular blockade, the trachea was intubated with the rigid bronchoscope, and the patients were ventilated with 100% oxygen via the Monsoon jet ventilator (Acutronic, Darmstadt, Germany). Settings were as follows: driving pressure 35 pounds per square inch, 80 breaths per minute, inspiratory time 40%, and 100% FiO2. The rigid suction catheter (attached to the gas analyzer with the apparatus described above) was introduced through the rigid bronchoscope (Figure 2) with the tip positioned at the main carina (Figure 3) to continuously measure AiO2. If AiO2 was less than 90%, ventilation was continued with 100% oxygen until the AiO2 reached >90%. Once this threshold was reached, ventilation was stopped and apnea was initiated. The time taken for the AiO2 to drop to 40% or lower in seconds was recorded. Jet ventilation was resumed with 100% oxygen when the AiO2 reached 40% or lower. The ASA does not recommend a specific end tidal FiO2 or AiO2 that is safe. We chose 40% as a threshold because it is within the range commonly targeted by institutions to attempt to reduce airway fire risk. The tip of the suction catheter/gas analyzer apparatus was then placed into the right main bronchus. Once the AiO2 at the new site reached 90% or greater, the apneic event described above was repeated. We recorded the time required for AiO2 to decrease to 40% or less. This was repeated a third time in the left main bronchus. During apnea, if the peripheral blood O2 saturation dropped below 90%, the study was stopped, and ventilation with 100% oxygen and the Monsoon jet ventilator was resumed.

Statistical analysis

Our study is a pilot study, and data were not available from prior studies to allow a priori sample size calculation. We evaluated our data using descriptive statistics. The comparison of average time to 40% at each site was evaluated using linear mixed-effects models with a random intercept for each participant. The correlation between pulmonary function tests and time to reach 40% in each location are evaluated with both Pearson's linear and Spearman's rank correlations.

## Results

**Figure 4:**
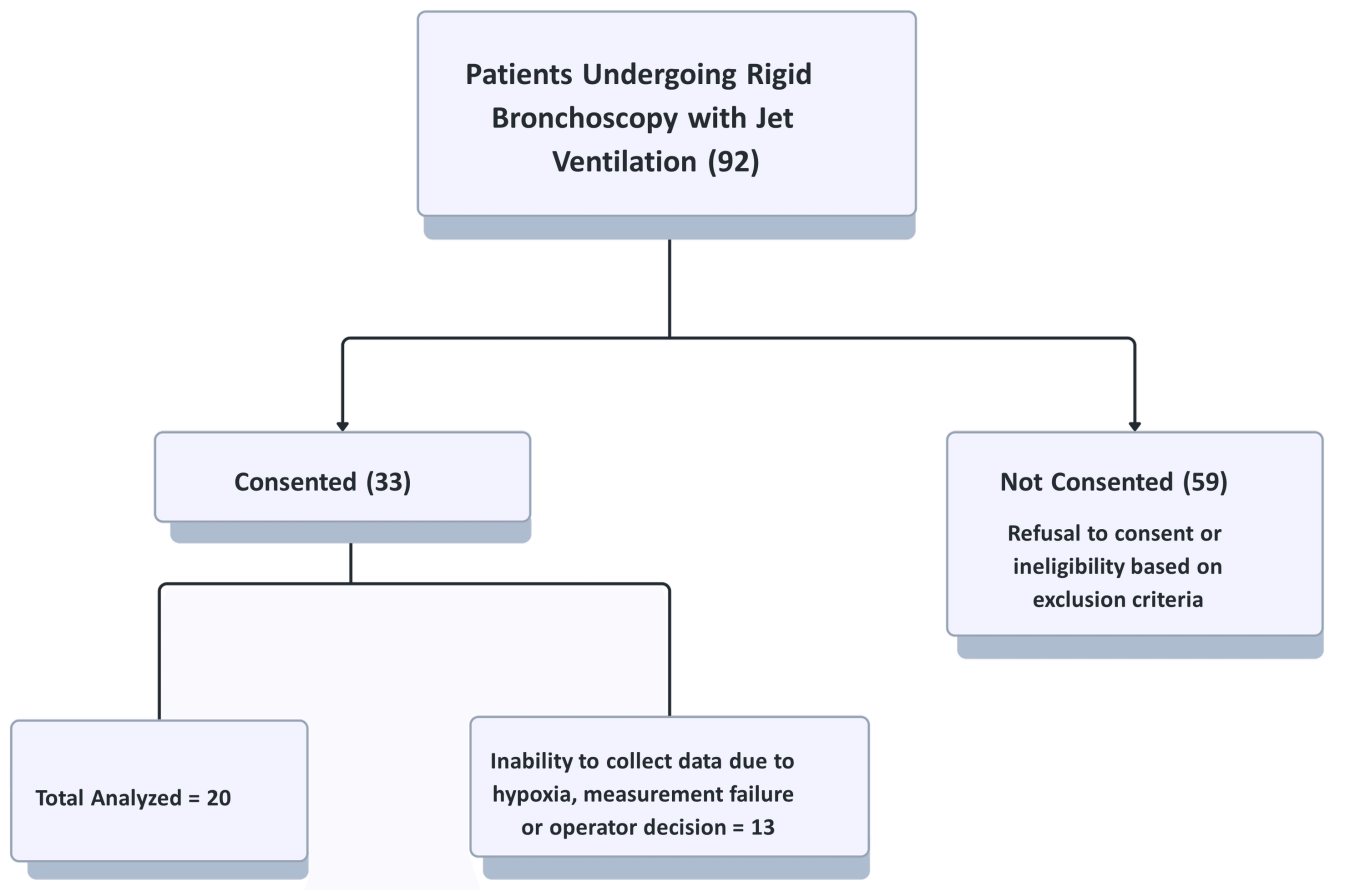
Patient recruitment flowchart

The patient recruitment flowchart is shown in Figure 4. Patient characteristics are described in Table 1. For 20 of 33 patients, we were able to collect complete data. For one patient, we were unable to reach an AiO2 of >90% at the beginning of the protocol. Data collection was stopped in six patients due to physician discretion because of clinical factors: either concern for poor tolerance of apnea or anatomic issues that made data collection not possible. In six patients, we had technical issues that precluded us from collecting data.

**Table 1:** Patient demographics. Continuous data is expressed as mean (standard deviation) or frequency (%) FVC: forced vital capacity; FEV1: forced expiratory volume in one second; TLC: total lung capacity; DLCO: diffusing capacity of the lungs for carbon monoxide

N	20
Sex: Male	12 (60%)
Age (y)	59.6 (8)
Disease	
Malignancy	4
Transplant	12
Other	4
Indication	
Bronchial stenosis	11
Tracheal stenosis	3
Tissue debulking	6
FVC (% pred)	75.2 (21.1)
FEV1 (% pred)	60.5 (24.2)
FEV1/FVC	62.7 (16)
TLC (% pred)	87.7 (26.5)
DLCO (% pred)	56.6 (24.7)

**Figure 5:**
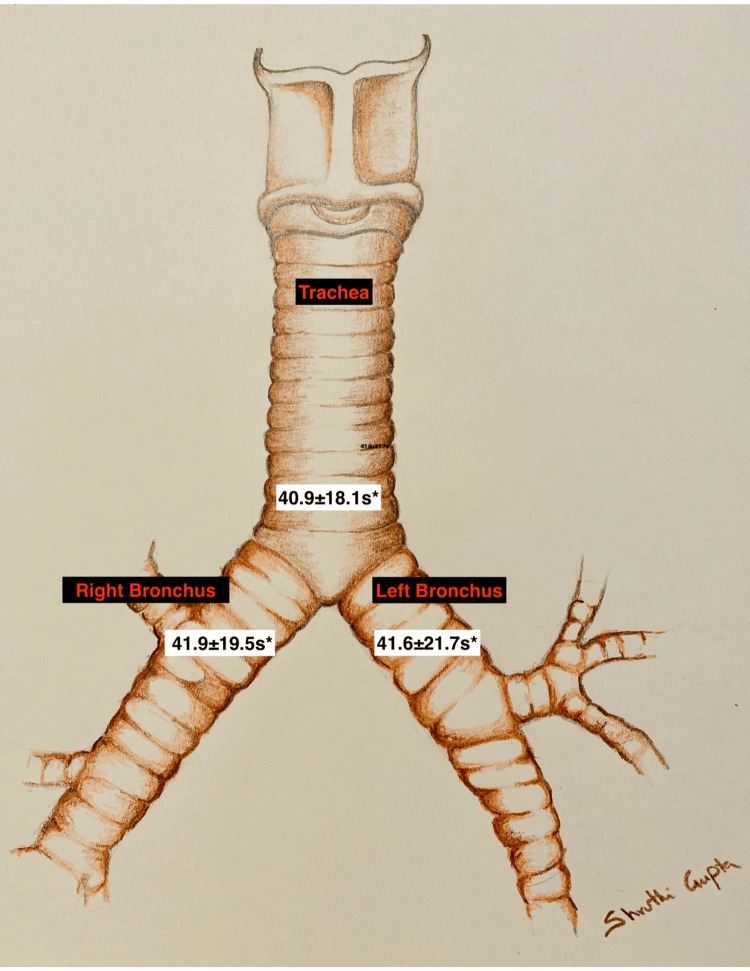
Time to end tidal oxygen concentration ≤40% (s) at the main carina, left bronchus, and right bronchus *: NS (not significant) Image Credit: Shruthi Gupta

The average length of apnea required to reach an AiO2 of 40% or less is shown in Figure 5. The time taken for the AiO2 to reach 40% from 90% was 40.9±18.1s for the distal trachea, 41.9±19.5s for the right mainstem bronchus, and 41.6±21.7s for the left mainstem bronchus. There was no statistically significant difference, whether sampling in the trachea or either bronchus. We found no relationship between time to goal AiO2 and age, sex, weight, or disease process. We found no correlation between time to 40% AiO2 and common preoperative pulmonary function test parameters, including total lung capacity, forced expiratory volume in one second, and diffusion capacity.

## Discussion

Real-time knowledge of airway oxygen concentration is a critical component in preventing fires during airway surgery. Our study demonstrates that (1) it is feasible to measure central airway oxygen concentration during rigid bronchoscopy and (2) from a baseline AiO2 of 90% or higher, after apnea of 40 seconds, a significant portion of our patients still had an AiO2 above 40%. This suggests that AiO2 remains above conventionally accepted levels for safe use of a heat source in the airway for an extended period of time during apnea. This is the first description of this method of monitoring AiO2 during rigid bronchoscopy.

Our findings are consistent with those described for end tidal oxygen concentration measured in the trachea of apneic patients undergoing direct laryngoscopy [8]. This suggests that, despite an open circuit, a prolonged period of apnea is often required before the oxygen concentration in the airway drops to "safe levels'' where the use of an ignition source in the airway would typically be considered [1-4]. The cause of this prolonged elevation of oxygen concentration in the central airways is multifactorial. The sedated and paralyzed patient likely has some decrease in oxygen consumption [9]. Additionally, despite the open circuit of a rigid bronchoscope, the lack of ongoing ventilation limits "washout" that could be achieved by continued ventilation at lower oxygen concentrations [10]. 

The apnea time required for AiO2 to reach goal levels was not associated with several commonly measured pulmonary function test parameters. This suggests a difficulty in the prediction of apnea efficacy in patients prior to rigid bronchoscopy. Our technique, by allowing close monitoring of AiO2 during the procedure, could allow more individualized decision-making for each patient during the procedure.

A limiting factor for the use of apnea during jet ventilation is hypoxemia. The decrease in airway oxygen concentration forms part of the cause, but derecruitment and atelectasis also may play a role [11]. In our study, one of 33 patients in our protocol developed hypoxemia prior to or shortly after reaching our goal "safe" oxygen concentration (AiO2 of 40%). This hypoxemia could affect the clinical adoption of prolonged periods of apnea to reach the optimal oxygen concentration. Alternatives, including jet ventilation with a mixture of oxygen and ambient air to allow for continued ventilation during the procedure, would need to be considered.

Our study had some limitations. Our sample size was relatively small, our protocol was conducted at one center, and the results may not be generalizable to other populations. Our study sample did not allow us to evaluate AiO2 behavior in relation to the position of specific segments of stenosis or to other common comorbidities. In individual patients, differences in response to induction medication or anesthesia could have affected our results. A longer or shorter length of pre-oxygenation could affect results. The gas sampling device on the Dräger Perseus A500 machine suctions around 200 ml of air/gas every minute. There is a possibility that gas sampling in the distal trachea or bronchi entrained room air into the airway, thereby diluting the AiO2 and shortening the length of apnea needed to reach 40% AiO2.

## Conclusions

Our data suggest that a prolonged apneic period may not reduce AiO2 to a level within commonly accepted parameters for reducing the risk of airway fire during jet ventilation with 100% oxygen using rigid bronchoscopy. Measurement of AiO2 at the site of a planned intervention prior to utilizing a heat source could provide helpful information during airway surgery with rigid bronchoscopy. Jet ventilation with a lower concentration of oxygen could preclude the need for prolonged apnea to reach goal AiO2 levels. This is an avenue for future research.
